# Consuming Two Eggs per Day, as Compared to an Oatmeal Breakfast, Decreases Plasma Ghrelin while Maintaining the LDL/HDL Ratio

**DOI:** 10.3390/nu9020089

**Published:** 2017-01-27

**Authors:** Amanda Missimer, Diana M. DiMarco, Catherine J. Andersen, Ana Gabriela Murillo, Marcela Vergara-Jimenez, Maria Luz Fernandez

**Affiliations:** 1 Department of Nutritional Sciences, University of Connecticut, Storrs, CT 06269, USA; amanda.missimer@uconn.edu (A.M.); diana.dimarco@uconn.edu (D.M.D.); ana.murillo_solis@uconn.edu (A.G.M.); 2 Department of Biology, Fairfield University, Fairfield, CT 06824, USA; candersen@fairfield.edu; 3 Department of Nutrition, Universidad Autonoma de Sinaloa, Culiacán 80040, Mexico; marveji@hotmail.com

**Keywords:** cholesterol, eggs, oatmeal, HDL-C, satiety, cardiovascular disease

## Abstract

Eggs contain high quality protein, vitamins, minerals and antioxidants, yet regular consumption is still met with uncertainty. Therefore, the purpose of this study was to compare the effects of consuming two eggs per day or a heart-healthy oatmeal breakfast on biomarkers of cardiovascular disease (CVD) risk and satiety measures in a young, healthy population. Fifty subjects participated in a randomized crossover clinical intervention; subjects were randomly allocated to consume either two eggs or one packet of oatmeal per day for breakfast for four weeks. After a three-week washout period, participants were allocated to the alternative breakfast. Fasting blood samples were collected at the end of each intervention period to assess plasma lipids and plasma ghrelin. Subjects completed visual analog scales (VAS) concurrent to dietary records to assess satiety and hunger. Along with an increase in cholesterol intake, there were significant increases in both low-density lipoprotein (LDL) and high-density lipoprotein (HDL) cholesterol following the egg consumption period (*p* < 0.01). However, there was no difference in the LDL/HDL ratio, a recognized biomarker of CVD risk, nor in the plasma glucose, triglycerides or liver enzymes, between diet periods. Several self-reported satiety measures were increased following the consumption of eggs, which were associated with lower plasma ghrelin concentrations (*p* < 0.05). These results demonstrate that compared to an oatmeal breakfast, two eggs per day do not adversely affect the biomarkers associated with CVD risk, but increase satiety throughout the day in a young healthy population.

## 1. Introduction

Cardiovascular disease (CVD) is currently the leading cause of death among adult men and women in the United States [1]. In order to prevent its development, beneficial diet and lifestyle habits should be implemented while young and healthy. Habitual breakfast consumption has been associated with a healthy lifestyle and good nutritional status, while studies have reported it to be predictive of lower body mass index (BMI), and inversely related to obesity later in life [2,3]. Whole eggs, which are typically consumed as a breakfast food, are inexpensive, readily available, and contain high quality proteins, essential vitamins and minerals, as well as the antioxidant carotenoids lutein and zeaxanthin, lecithin, choline and cholesterol [4]. In the past, whole egg consumption has been controversial, due to the cholesterol content of eggs (~185–200 mg/egg), although this amount of dietary cholesterol has been long since identified as a non-significant independent risk factor for CVD [5]. Currently, the American Heart Association is recommending egg whites without the yolks as a heart-healthy source of protein [6], despite the removal of the 300 mg/day limit of dietary cholesterol in the 2015–2020 Dietary Guidelines for Americans [7]. The negative connotation associated with dietary cholesterol has been challenged by numerous studies demonstrating the lack of increased biomarkers associated with risk of CVD and the beneficial effects of daily egg consumption [8,9,10]. Whole egg consumption studies have not only resulted in the absence of increases in factors associated with CVD risk but also in improved markers of chronic inflammation, glucose intolerance, and insulin resistance in several distinct populations: children, individuals with metabolic syndrome and people with diabetes [11,12,13,14]. A previous study showed that the consumption of one egg per day reduces insulin resistance and contributes to higher weight loss associated with increased satiety [15]. In several studies, when compared to an isoenergetic, equal-weight breakfast, an egg-based breakfast has been shown to increase satiety and decrease energy intake [13,16,17]. Satiety, or the feeling of satisfaction, can be measured objectively, by biochemical measures, or subjectively by self-report. Increased satiety can lead to changes in nutrient intake patterns, which can have favorable roles in the reduction of CVD risk [18,19,20]. Specifically, ghrelin, a growth hormone—releasing peptide secreted by the stomach in a fasted state, promotes food intake and has roles in energy balance and weight maintenance, as well as proposed roles in various body systems [21]. The regulation of this hormone is effected by both chronic energy balance and acute feeding. It is a marker for increased appetite, and is associated with a counter hormone, leptin, which has been implicated in the regulation of adipocyte homeostasis [18,22,23]. Decreased levels of ghrelin have been related to decreased appetite and can increase weight loss [24,25].

For the purpose of this study, whole egg consumption was compared to oatmeal consumption. Oatmeal is also a good source of fiber, B vitamins, iron, magnesium, and selenium, and an American Heart Association “certified heart healthy food” [26]. The main benefit of oatmeal for CVD protection is the ability to lower low-density lipoprotein cholesterol (LDL-C), which can be attributed to its soluble fiber content [27]. The main objective of this study was to investigate the effects of two different breakfasts on anthropometrics, dietary patterns, lipid profile, and satiety in a healthy population in order to evaluate the biomarkers of CVD risk and effect on appetite. We hypothesized that compared to daily consumption of oatmeal, the intake of two eggs per day would not result in changes of CVD risk biomarkers, but would increase satiety in a young, healthy population.

## 2. Materials and Methods

### 2.1. Experimental Design

Fifty healthy individuals (26 females and 24 males) between the ages of 18–30 years were recruited to participate in an 11-week, randomized, crossover, intervention study. Participants were mainly college students recruited from the University of Connecticut, Storrs, CT and young professionals from local surrounding communities. Based on the standard deviation from our previous studies and using a *Z* value of 1.96 (95% confidence interval) we estimated that 40 subjects would be sufficient to observe differences in plasma high-density lipoprotein cholesterol (HDL-C) between groups [11,12,15]. We recruited 50 subjects to allow for attrition. The study took place between August–December 2014.

Participants signed a written, informed consent form prior to any measures or experimental procedures. Inclusion criteria included age (18–30 years), body mass index (BMI) (18.5–29.9 kg/m^2^) and willingness to consume the intervention foods daily.

Exclusion criteria included plasma triglycerides (TG) > 500 mg/dL, total cholesterol (TC) > 240 mg/dL or glucose (GLU) > 126 mg/dL. Current or previous history of liver disease, renal disease, diabetes, cancer, endocrine disorders, metabolic disorders, stroke or heart disease, taking glucose lowering drugs or supplements, current pregnancy or lactation, BMI ≥ 30 kg/m^2^, blood pressure > 140/90, egg or gluten allergy/sensitivity or celiac disease. All analysis was performed blinded to breakfast allocation. All visits took place in the Nutritional Sciences Department at the University of Connecticut, Storrs, CT. This study was approved by the Institutional Review Board at the University of Connecticut (protocol #H14-032). This trial is registered at ClinicalTrials.gov as NCT02181244.

### 2.2. Dietary Intervention

Enrolled participants were randomly allocated to consume either two eggs per day (EGGS) or one packet of oatmeal per day (OATS) for four weeks (Big Y Foods, Inc., Springfield, MA, USA). Following a three-week washout, participants crossed over to the alternate intervention food for four weeks. Each daily serving of two eggs contained 370 mg cholesterol, 0 g carbohydrate, 12 g protein, 10 g fat, 0 g fiber and 140 calories. Each daily serving of one packet of oatmeal had 0 mg cholesterol, 0–14 g carbohydrate, 3–4 g protein, 1.5–2 g fat, 3 g fiber and 100–160 calories depending on the flavor choice, of which there were five. Participants were instructed to consume the intervention foods as the first meal of the day, and were allowed to add vegetables, meat, cheese, syrup, yogurt, etc. to their breakfast intervention food, if desired. Participant compliance was monitored daily by self-report and bi-weekly visits to our laboratory for product pickup. During the intervention and washout periods, participants were asked to avoid consuming whole eggs or foods containing predominately eggs or oats. Aside from treatment, habitual dietary intake, exercise, medication usage, and supplement intake was maintained throughout the study.

### 2.3. Blood Collection and Processing

After a 12 h overnight fast, blood was drawn at baseline, and at the end of each dietary period. Whole blood was collected from participants in EDTA and SST blood collection tubes and was centrifuged at 2000× *g* for 20 min at 4 °C. Plasma and serum were collected and aliquots were placed at −80 °C for storage.

### 2.4. Anthropometrics, Blood Pressure, Waist Circumference, and Dietary Intake

Anthropometric measures of weight, height, BMI, and waist circumference (WC), and dietary intake were collected from participants at the end of each dietary period. Weight was measured to the nearest 0.1 kg, height to the nearest centimeter, and BMI was calculated. Blood pressure (BP) was measured with an automated blood pressure monitor (Omron, Healthcare Inc., Bannockburn, IL, USA) after a 5 min rest. The average of three separate recordings is reported. WC was measured on bare skin, at the top of the iliac crest to the nearest 0.1 cm. The average of 3 separate recordings is reported. Subjects were responsible for recording all food and beverages consumed, for two non-consecutive weekdays and one weekend day. Dietary intake was analyzed using Nutrition Data System for Research (NDSR) 2014 (Nutrition Coordinating Center, University of Minnesota) to quantify macronutrient, micronutrient and carotenoid intake, glycemic index, and glycemic load.

### 2.5. Plasma Lipids, Glucose, and Liver Enzymes

Fasting plasma samples from the end of each intervention period were analyzed for TC, HDL-C, TG, GLU, alanine aminotransferase (ALT), and aspartate aminotransferase (AST) levels. Samples were measured using an automated clinical chemistry analyzer (Cobas c111, Roche Diagnostics, Indianapolis, IN, USA), LDL-C was estimated using the Friedewald equation [28].

### 2.6. Measures of Satiety

Satiety was analyzed by subjective (satiety visual analog (VAS) scales) and objective (plasma ghrelin) measurements. Participants completed corresponding VAS on the same days as the three-day dietary intake records [15]. Satiety scales were completed prior to breakfast, lunch and dinner. Each VAS questionnaire was composed of eight open-ended questions regarding hunger, satisfaction, fullness, satiety and taste preference for savory, salty, sweet, or fatty foods. The VAS was a 10 cm line, ranging from “not at all” to “yes very much”. Participants marked along the line indicating their feelings, which was then assigned a quantifiable value by measuring from the beginning of the line to where the participant had marked. Values were recorded to the tenth position for analysis. To measure ghrelin, fasting plasma from the end of each intervention period was acidified with 1 M hydrochloric acid to retain stability at −80 °C. Total ghrelin was quantified using a sandwich ELISA kit, (Mercodia AB, Uppsala, Sweden).

### 2.7. Statistical Analysis

All statistical analyses were performed using SPSS version 22. Paired *t*-tests were used to assess differences between dietary treatments and baseline values were used as a covariate. Data are reported as mean ± SD unless noted otherwise. *p* < 0.05 was considered significant.

## 3. Results

### 3.1. Study Flow Chart

**Figure 1:**
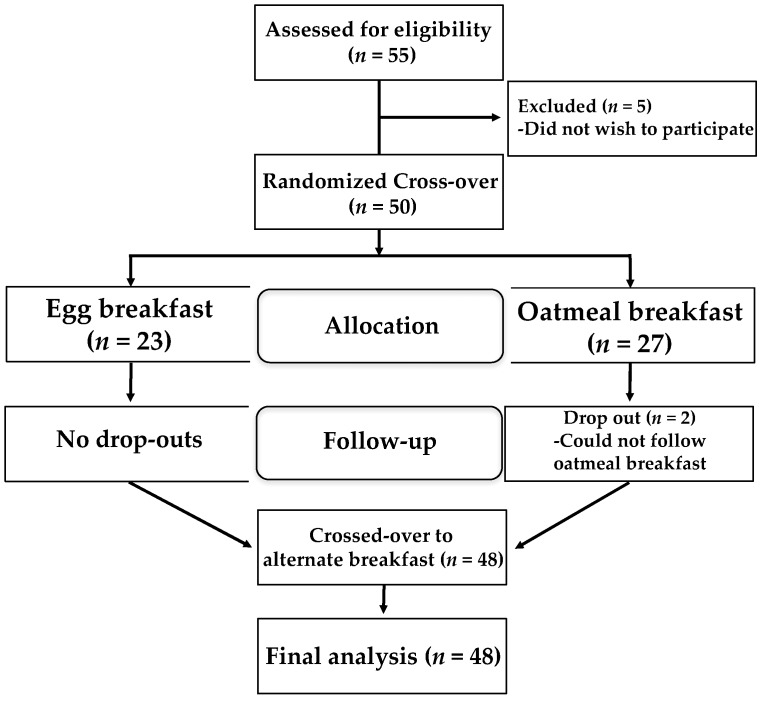
Flow chart of the study design. Forty-eight of 50 participants completed the study, as they did not meet requirements for oatmeal consumption during that arm of the intervention.

Of the 55 potential participants screened, five were excluded due to a personal decision not to participate. During the first phase of the study, two participants dropped out due to failed compliance while consuming oatmeal; 48 participants completed the study (Figure 1).

### 3.2. Baseline Characteristics

Characteristics of participants at baseline are shown in Table 1. The average age of participants was 23.3 years with a distribution of 26 females and 24 males. Baseline anthropometrics were within healthy ranges, with an average BMI of 23.2, a WC of 81.3 and an average BP of 112/72 mmHg. Participants had a healthy lipid profile, with average TC < 3.9 mmol/L, LDL-C < 1.9 mmol/L, TG < 0.8 mmol/L, and HDL > 1.7 mmol/L. The average fasting GLU < 5.1 mmol/L and C-reactive protein (CRP) of 0.2 mg/L indicated no significant glucose sensitivity or inflammation, respectively, at baseline.

### 3.3. Dietary Intake

While there was no significant difference in energy intake between treatment periods, following the EGGS period, the percentage of calories from protein and fat increased (*p* < 0.001), while the percentage of calories from carbohydrates decreased (*p* < 0.001), (Table 2). The intake of both saturated fatty acids (SFA) and monounsaturated fatty acids (MUFA) was increased with egg consumption (*p* < 0.001), while there was no difference in the intake of polyunsaturated fatty acids (PUFA) between treatment groups. As expected, the dietary cholesterol intake was increased during the EGGS period (*p* < 0.001). Comparatively, the total and soluble fiber intake was significantly increased during the OATS period (*p* < 0.05). Despite egg yolks serving as a bioavailable source of lutein and zeaxanthin [29], there was no difference in the dietary intake of the carotenoids lutein and zeaxanthin between the EGGS and OATS periods. Similarly, no changes in the glycemic index were observed between treatments, whereas the glycemic load was decreased during the EGGS period (*p* < 0.05) (Table 2).

### 3.4. Anthropometrics, Blood Pressure, Plasma Lipids, Glucose, and Liver Enzymes

There were no differences in the anthropometric measures of WC, BMI, and systolic or diastolic BP between the two breakfasts. Following egg intake there was an increase in TC, LDL-C, and HDL-C (*p* < 0.05), with no change in the LDL-C/HDL-C ratio. There were no significant differences in TG, GLU, ALT or AST when participants were consuming eggs or oatmeal for breakfast (Table 3).

### 3.5. Measurements of Satiety: VAS and Plasma Ghrelin

**Figure 2:**
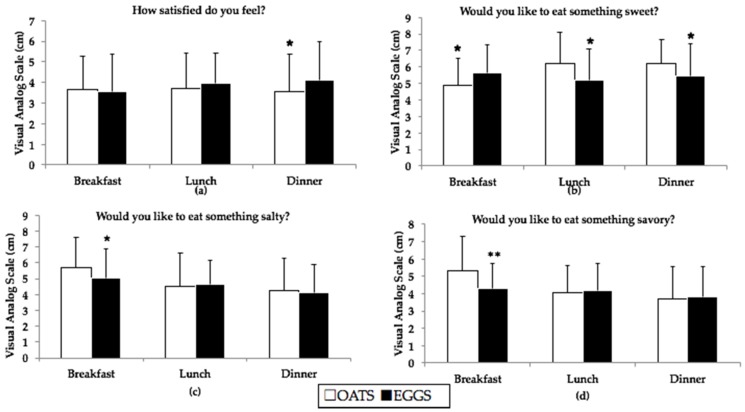
Results from satiety VAS completed prior to each meal during OATS (white bars) and EGGS (black bars) periods; *n* = 48. (**a**) Participants consuming two eggs per day felt more satisfied prior to eating dinner; (**b**) Participants consuming two eggs per day wanted something sweet for breakfast as compared to lunch and dinner; (**c**) Participants consuming two eggs did not prefer something salty as compared to oatmeal; (**d**) Participants wanted something less savory following the consumption of two eggs per day; * different from oatmeal at *p* < 0.05 and ** different from oatmeal at *p* < 0.01.

The analysis of VAS revealed that participants consuming eggs for breakfast felt more satisfied prior to consuming dinner than participants consuming oatmeal, suggesting a prolonged effect of egg intake on satiety throughout the day (Figure 2a). Participants consuming eggs felt more of a desire to consume something sweet for breakfast, then less of a desire for something sweet prior to lunch and dinner (Figure 2b). Finally, participants consuming eggs felt less of a desire to consume something salty or savory for breakfast, as compared to participants consuming oatmeal for breakfast (Figure 2c,d).

**Figure 3:**
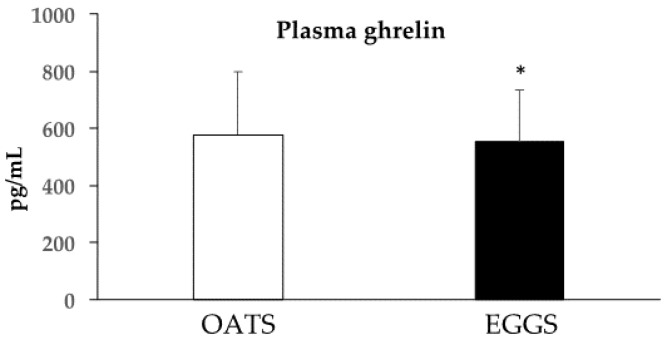
Fasting plasma ghrelin concentrations for subjects after the oatmeal (577.8 ± 219.7 pg/mL) and eggs (553.0 ± 181.5 pg/mL) periods, * *p* < 0.05. Participants (*n* = 48) had lower circulating total ghrelin following consumption of two eggs.

**Figure 4:**
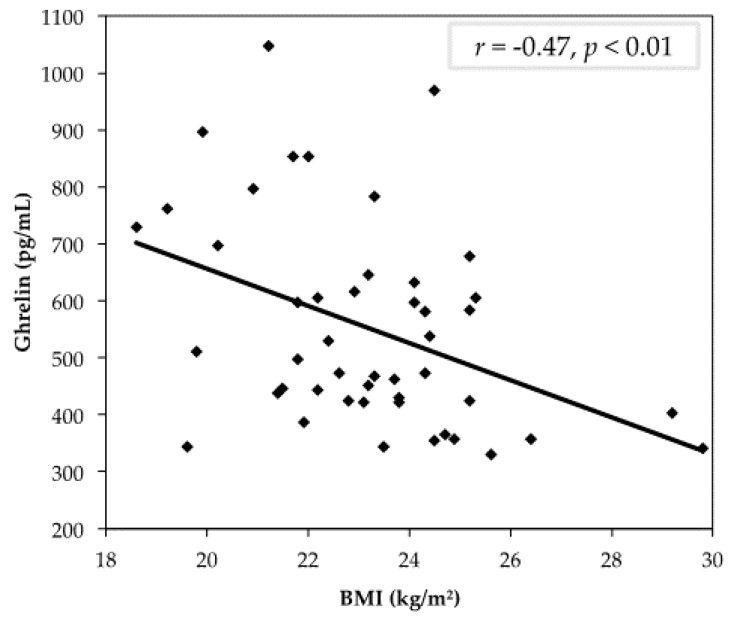
Correlation between fasting plasma ghrelin and body mass index (BMI) following the consumption of two eggs for breakfast; participants (*n* = 48), *r* = −0.47, *p* < 0.01.

We further aimed to examine an objective measure of satiety. Following four weeks of eggs consumption for breakfast, fasting plasma ghrelin was significantly decreased as compared to an oatmeal breakfast, indicating less feelings of hunger upon waking as compared to an oatmeal breakfast (Figure 3). A negative correlation was identified between ghrelin and BMI, indicating an association between the biological indication of hunger and body weight (Figure 4).

## 4. Discussion

Despite the recent removal of the dietary cholesterol intake limit [30], habitual egg consumption remains controversial and misunderstood by the general public. Therefore, the purpose of this study was to determine whether an egg-based breakfast as compared to a certified heart-healthy breakfast of oatmeal would increase the biomarkers associated with CVD risk and affect appetite in a young, healthy population. Although there was an increase in both LDL-C and HDL-C, we found the consumption of two eggs per day as compared to an oatmeal breakfast did not increase the LDL-C/HDL-C ratio, while increasing satiety as measured by fasting plasma ghrelin and VAS.

The impact of dietary cholesterol on plasma lipids varies among individuals, potentially due to variations in the negative feedback regulation of endogenous cholesterol production in response to cholesterol intake [31]. In this study, the consumption of two eggs per day did raise TC levels, corresponding to the increased intake of dietary cholesterol as compared to oatmeal. Notably, egg-induced increases in TC are related to concurrent increases in both LDL-C and HDL-C, which has been observed in previous egg studies [12,32,33]. The maintenance of the LDL-C/HDL-C ratio, an accepted CVD prediction model, does not impact CVD risk, which, in contrast to previous epidemiological studies that associated egg consumption with increased LDL-C only, then extrapolated that data to an increased risk of CVD with egg intake [34].

This concurrent increase in HDL-C may be suggestive of improved reverse cholesterol transport (RCT) because it is indicative of greater amounts of cholesterol being removed from the tissues [35]. It is hypothesized that the phospholipids from eggs are involved in this modulation of RCT by impacting the HDL size and function, as well as the expression of hepatic HDL uptake receptors [36]. In a human clinical trial, ex vivo cholesterol efflux, the critical first step of RCT, was found to be increased following egg consumption [37]. Two large meta-analyses have reported no association between regular egg consumption and the risk of CVD, myocardial infarction or stroke in the general population [38,39]. Thus, although TC, LDL-C and HDL-C levels were increased following egg consumption, regulatory mechanisms may have compensated for the added dietary cholesterol and biomarkers of CVD risk remained unchanged in this population.

Our results reported the increased consumption of protein and fat throughout the day, with no impact on the total energy intake or change in body weight following the consumption of two eggs at breakfast. High-protein diets have been found to increase energy expenditure, and promote a negative energy balance [17]. In a study in healthy individuals who consumed low- or high-protein diets, with sustained carbohydrate consumption (50%), researchers found a decrease in appetite and caloric intake with high-protein intake. In our study, a decrease in hunger and an increase in fullness were seen between weeks 3 and 4 of the isocaloric high-protein diet; however, a decrease in caloric intake was sustained for the ad libitum high-protein leg of the study. This correlated with increased plasma ghrelin over time. Although this study did not reduce the amount of carbohydrates in the diet, we found similar results to a high-protein diet with a decreased carbohydrate intake as well [18]. The glycemic load is an accurate measure of the degree to which carbohydrate-containing foods will affect plasma glucose levels [40]. Reductions in glycemic load have previously been associated with carbohydrate-restricted diets and egg consumption [41]. The reduction in carbohydrates following the consumption of eggs is the likely cause of the reduction in the glycemic load, which has been implicated in the control of glucose and lipid metabolism [42].

Protein increases satiety and thus may explain the decrease in ghrelin levels. In a study investigating the effect of protein intake on the plasma ghrelin concentration, there was a significant reduction in ghrelin over time when consuming higher-protein diets compared to carbohydrate diets [18]. A study in children and adolescents evaluated satiety at lunchtime following either an egg or bagel breakfast [43]. There were no significant differences in dietary intake at lunchtime in either group, according to dietary intake analysis and VAS. However, the peptide YY (PYY) was increased following egg consumption, which is also suggestive of increased satiety. This increase was observed often after consuming foods higher in protein and fat. The authors attributed the lack of correlation between dietary consumption and ghrelin levels to the children’s ability to self-regulate food intake better than adolescents and adults [44]. Conversely, a study investigating the impact of macronutrient intake on levels of cortisol, which stimulates food intake, found decreased levels throughout the day following the intake of protein and fat as compared to carbohydrates [45]. Equally, the intake of carbohydrates for breakfast caused an increase in the cortisol concentration, which may be related to increased feelings of hunger throughout the day. Eating comfort food, high in sugar, is proposed to cause stress that further increases cortisol concentrations throughout the day [45]. Ghrelin is down-regulated in positive energy balance, and up-regulated in negative energy balance, in healthy-weight individuals [46]. Tschop et al. [43,47] also reported a negative correlation between fasting ghrelin and percent body fat. In our study, we found similar results, with those individuals with higher BMIs having the lowest plasma ghrelin. This can be attributed to a negative feedback mechanism, in which the body is attempting to maintain energy balance homeostasis and the regulation of feeding [48].

Following egg consumption, we likewise observed decreased circulating ghrelin. This may be related to the increased protein intake as a result of egg intake. Egg protein has the highest biological value of any other protein at an 88–95/100 rating, indicating the presence of all amino acids, both dispensable and indispensable. High-biological-value proteins have been suggested to delay gastric emptying and to have a stronger effect on decreasing post-prandial ghrelin concentrations [24]. Ghrelin also follows a diurnal pattern, dipping around 2 p.m. and steadily increasing as the night continues, which can be implicated in hunger upon waking [47]. From comparing our results, in which we observed decreased plasma ghrelin and increased satisfaction at dinnertime with egg consumption, we can hypothesize that eggs play a long-term role in satiety. In a study investigating the effect of protein intake on plasma ghrelin concentrations, there was a significant reduction in ghrelin over time when consuming higher-protein diets as compared to carbohydrate diets [49]. This may be due to the satiating effects of protein and the impact of easy digestion. In the case of egg consumption, the high biological value helps with the digestibility and improved satisfaction at dinnertime compared to the oatmeal breakfast [50].

There are several limitations to this study including the use of whole foods for the intervention, which does not allow the participants to be blinded to the specific breakfast. However, any bias over the foods consumed was removed since no expectations of the data were shared with the participants. Another limitation would be ghrelin measurement, which was assessed prior to consuming breakfast only. The post-prandial assessment of ghrelin would have strengthened the results found by VAS analysis. While appetite was affected following the consumption of eggs, there were no observed differences in energy intake. This could be considered a limitation, because though satiety was improved, this may not be considered an effective treatment for weight loss. There were also some strengths to this study including the use of whole foods, as the results can be easily extrapolated to the general population, because these foods are normally consumed for breakfast. The assessment of satiety via VAS and the plasma measurement of ghrelin were also strengths of this study in evaluating and correlating hunger and the desire to eat throughout the day.

The intake of two eggs per day as compared to an oatmeal breakfast promoted a shift in dietary intake patterns, did not lead to an increase in biomarkers associated with CVD, and resulted in both subjective and objective measures of satiety in a healthy population. The results of the study are important to confirming eggs as a healthy habitual breakfast food with additional benefits of increased satiety throughout the day.

## Figures and Tables

**Table 1:** Baseline characteristics of subjects ^1^.

Parameter	Values
Age (years)	23.3 ± 3.1
Gender (*n* = F/M)	26/24
BMI (kg/m^2^)	23.2 ± 2.1
WC (cm)	81.3 ± 6.5
Systolic BP (mmHg)	112.1 ± 12.4
Diastolic BP (mmHg)	72.7 ± 7.0
Total Cholesterol (mmol/L)	3.9 ± 0.7
LDL-C (mmol/L)	1.9 ± 0.6
HDL-C (mmol/L)	1.7 ± 0.5
LDL-C/HDL-C	1.2 ± 0.07
Triglycerides (mmol/L)	0.9 ± 0.04
Glucose (mmol/L)	5.1 ± 0.3
CRP (mg/dL)	0.2 ± 0.8

^1^ Values are presented as mean ± SD; *n* = 50.

**Table 2:** Dietary intake during eggs and oatmeal periods ^1^.

Parameter	Eggs	Oatmeal
Weight (kg)	68.2 ± 11.2	68.2 ± 11.1
Energy (kcal)	1937 ± 630	2016 ± 1461
Protein (%)	19.2 ± 4.4	17.6 ± 4.1
Carbohydrate (%)	41.4 ± 6.2 **	48.9 ± 8.2
Total Fat (%)	37.2 ± 5.1 **	32.2 ± 6.7
SFA (g/day)	27.3 ± 11.9 **	21.1 ± 8.1
MUFA (g/day)	30.4 ± 11.6 **	23.2 ± 9.6
PUFA (g/day)	16.6 ± 7.4	15.7 ± 11.5
Total Fiber (g/day)	18.3 ± 7.0	20.9 ± 11.4
Soluble Fiber (g/day)	5.6 ± 2.6 *	7.0 ± 4.0
Insoluble Fiber (g/day)	12.7 ± 7.4	13.9 ± 8.0
Cholesterol (mg/day)	546.1 ± 96.6 **	173.1 ± 90.6
Lutein + Zeaxanthin (µg/day)	2820 ± 3443	2327 ± 3997
Glycemic Index	59.0 ± 5.9	59.9 ± 6.0
Glycemic Load	109.9 ± 42.4 *	122.6 ± 49.1

^1^ Values are presented as mean ± SD; *n* = 48; * *p* < 0.025, ** *p* < 0.001.

**Table 3:** Anthropometrics, blood pressure, plasma lipids, glucose, and liver enzymes after the eggs and oatmeal breakfasts ^1^.

Parameter	Eggs	Oatmeal
BMI (kg/m^2^)	23.2 ± 2.2	22.7 ± 2.6
WC (cm)	81.9 ± 6.4	82.6 ± 6.6
Systolic BP (mmHg)	110.3 ± 10.1	111.2 ± 11.7
Diastolic BP (mmHg)	74.4 ± 6.4	73.4 ± 6.3
Total cholesterol (mmol/L)	4.2 ± 0.7 *	4.0 ± 0.7
LDL cholesterol (mmol/L)	2.1 ± 0.7 *	1.9 ± 0.6
HDL cholesterol (mmol/L)	1.71 ± 0.48 *	1.62 ± 0.47
LDL-C/HDL-C	1.35 ± 0.62	1.30 ± 0.56
Triglycerides (mmol/L)	0.89 ± 0.37	0.91 ± 0.41
Glucose (mmol/L)	5.1 ± 0.4	5.0 ± 0.4
ALT (U/L)	18.2 ± 7.9	17.6 ± 6.0
AST (U/L)	24.5 ± 7.8	24.5 ± 8.8

^1^ Values are presented as mean ± SD; *n* = 48; * *p* < 0.025.
